# The Epigenetic Faces of ULTRAPETALA1

**DOI:** 10.3389/fpls.2021.637244

**Published:** 2021-02-25

**Authors:** Diego Ornelas-Ayala, Adriana Garay-Arroyo, Berenice García-Ponce, Elena R. Álvarez-Buylla, María de la Paz Sanchez

**Affiliations:** ^1^Laboratorio de Genética Molecular, Epigenética, Desarrollo y Evolución de Plantas, Instituto de Ecología, Universidad Nacional Autónoma de México, 3er Circuito Ext. Junto a J. Botánico, Ciudad Universitaria, UNAM, Mexico City, Mexico; ^2^Centro de Ciencias de la Complejidad (C3), Universidad Nacional Autónoma de México, Mexico City, Mexico

**Keywords:** ULTRAPETALA1, TrxG, PcG, ATX1, Molecular epigenetic switch, Arabidopsis

## Abstract

ULTRAPETALA1 (ULT1) is a versatile plant-exclusive protein, initially described as a trithorax group (TrxG) factor that regulates transcriptional activation and counteracts polycomb group (PcG) repressor function. As part of TrxG, ULT1 interacts with ARABIDOPSIS TRITHORAX1 (ATX1) to regulate H3K4me3 activation mark deposition. However, our recent studies indicate that ULT1 can also act independently of ATX1. Moreover, the ULT1 ability to interact with transcription factors (TFs) and PcG proteins indicates that it is a versatile protein with other roles. Therefore, in this work we revised recent information about the function of Arabidopsis ULT1 to understand the roles of ULT1 in plant development. Furthermore, we discuss the molecular mechanisms of ULT1, highlighting its epigenetic role, in which ULT1 seems to have characteristics of an epigenetic molecular switch that regulates repression and activation processes via TrxG and PcG complexes.

## Introduction

In multicellular organisms, epigenetic regulation plays crucial roles for the correct deployment of developmental programs and for the establishment of cell fates. Epigenetic mechanisms include post-translational histone modifications (PHM) that modulate chromatin structure to regulate gene expression. The trithorax group (TrxG) is an epigenetic protein complex able to regulate transcriptional activation through trimethylation of lysine 4 and 36 of histone H3 (H3K4me3 and H3K36me3) as well as other associated PHMs (Schuettengruber et al., 2011). TrxG proteins are those that belong to complexes counteracting of polycomb group (PcG) repressive activity at the same set of target genes (Grimaud et al., 2006); however, other proteins that act together with TrxG on PcG or non-PcG target genes are also considered TrxG (Schuettengruber et al., 2007).

**FIGURE 1:**
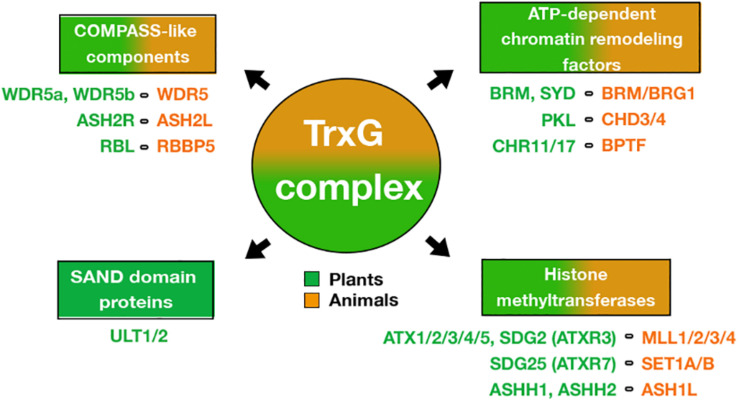
Factors of the TrxG complex. Different proteins of the TrxG animal complex that are conserved in plants such as (i) ATP-dependent chromatin-remodeling factors, (ii) SET domain-containing proteins that catalyze histone methylation, and (iii) COMPASS-like proteins. It is noteworthy that plant TrxG includes SAND-domain proteins that are not conserved in animals. The list of names below of each TrxG component shows the proteins described in Arabidopsis (green color) and their mammalian counterparts (orange color).

In plants, TrxG participates in different developmental processes from embryogenesis to floral development, regulating gene expression of several transcription factors (TFs) involved in stem cell maintenance, cell fate identity, and cell proliferation and differentiation (Sanchez et al., 2015; Fletcher, 2017). The plant TrxG complex has been identified by homology to known TrxG proteins in animals or by genetic characterization based on their ability to counteract PcG mutant phenotypes (Fletcher, 2017). In this regard, SET histone methyltransferases (HMTs) of MLL and SET families, COMPASS-like proteins such as WDR5, ASH2L and RBBP5, and ATP-dependent chromatin-remodeling factors such as BRM, CHD and BPTF, have been described in plants (Avramova, 2009; Schuettengruber et al., 2011; Sanchez et al., 2015) (Figure 1). In *Arabidopsis thaliana* (hereafter Arabidopsis), the main HMTs of TrxG that catalyze the H3K4me3 mark are the ARABIDOPSIS TRITHORAX1 (ATX1) and the ARABIDOPSIS TRITHORAX-RELATED 3/SETDOMAIN GROUP 2 (ATXR3/SDG2) (Alvarez-Venegas et al., 2003; Berr et al., 2010; Guo et al., 2010; Chen et al., 2017), although until now, only ATX1 has been found to form a complex within the core of Arabidopsis COMPASS-like complex described (Jiang et al., 2011). Interestingly, it has been reported that the plant TrxG group includes a unique protein named ULTRAPETALA1 (ULT1) (Figure 1), whose structure differs from all TrxG components reported in animals and yeast. ULT1 has been defined as a TrxG factor by counteract PcG silencing and by its physical interaction with ATX1 (Carles and Fletcher, 2009; Pu et al., 2013). However, our recent study indicates that ULT1 can act independently of ATX1, in a tissue-specific fashion (Ornelas-Ayala et al., 2020). Moreover, the interactions of ULT1 with PcG proteins (Xu et al., 2018) suggest other roles of ULT1 as well. Therefore, here we review recent information on the structure of the ULT1 protein, its interactions with other proteins, and its gene targets, as well as the phenotypic analysis of loss-of-function mutants to understand the roles of ULT1 in plant development. Furthermore, we discuss the molecular mechanisms in which ULT1 is involved, as well as its possible function as an epigenetic molecular switch that regulates repression and activation processes via TrxG and PcG complexes.

## What the ULT1 Structure Reveals About Its Function

**FIGURE 2:**
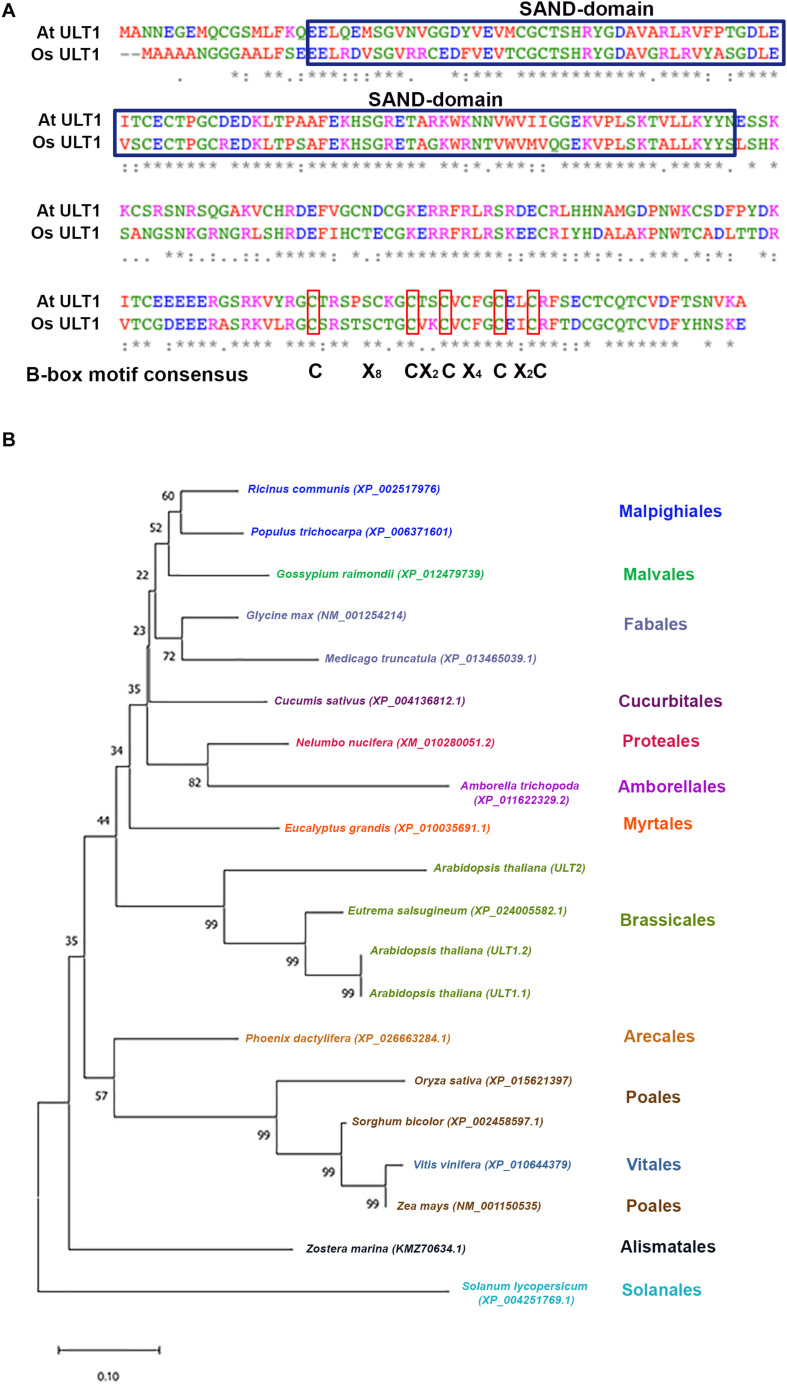
ULTRAPETALA1 is conserved in different angiosperm species. **(A)** The SAND-domain and B-box motif of ULT1 from *Arabidopsis thaliana* and its alignment with ULT1 from *Oryza sativa*. The asterisks show identical residues; colons (:) and periods (.) show residues with strongly and weakly similar properties, respectively. The blue boxes show the SAND-domain and the red boxes represent the B-box consensus motif. **(B)** Phylogenetic analysis generated using the neighbor-joining method based on the ULT1 protein sequence of selected plant species. Numbers at nodes represent bootstrap percentages based on 10,000 samplings. The scale bars represent 0.1 substitutions per site.

In Arabidopsis, ULT1 has been described as a SAND (named after Sp100, AIRE, NucP41/75, DEAF-1) domain protein that also contains a B-box motif (Figure 2A), a motif that seems to be important for protein-protein interaction (Torok and Etkin, 2001; Carles et al., 2005; Khanna et al., 2009). In the case of OsULT1 from *Oryza sativa*, it has been shown that is important for its multimerization (Roy et al., 2019). Meanwhile, the SAND domain has a DNA-binding function (Bottomley et al., 2001), and it is conserved in plants and animals in vast combinations with other protein domains on the Viridiplantae and metazoan lineages. The Clorophyta lineage contains a single-SAND domain protein RegA, whereas in the Embryophyte lineage only ULT and ATX3 (ARABIDOPSIS THRITHORAX3) proteins and its paralogs contain a SAND domain (Kirk et al., 1999; Nedelcu, 2019). In ULT proteins, the SAND domain is unique, whereas in ATX3, it appears in combination with the SET-like and PHD domains (Nedelcu, 2019). The SAND domain in combination with other protein domains has also been related to chromatin interactions and transcriptional regulation. For instance, AIRE (Autoimmune Regulator) is capable of interacting with chromatin through its PHD domain. AIRE binds specifically unmethylated H3K4 residues and it is proposed that this binding is important for its function as a transcriptional activator (Org et al., 2008). Moreover, the AIRE protein can associate with DNA transcriptional control elements and factors involved in pre-mRNA processing (Abramson et al., 2010) and also can be acetylated by the CBP (CREB Binding Protein) and the p300 histone acetyltransferases to enhance its transactivation activity (Saare et al., 2012). Therefore, the SAND domain is a DNA-binding module characteristic of chromatin-dependent transcriptional regulation. In fact, by *in vitro* assays, it has been shown that the SAND domain of human DEAF-1 (Deformed Epidermal Autoregulatory Factor-1) homolog recognizes the 5′-TTCG-3′ sequence (Bottomley et al., 2001). This sequence differs from what has been reported in plants, where the SAND domain of recombinant OsULT1, has affinity for the 5′-GAGAG-3′ sequence (Roy et al., 2019).

Most of the SAND domain proteins of the different lineages are involved in developmental processes such as cell proliferation, cell differentiation, tissue homeostasis and organ formation (Nedelcu, 2019). For instance, in the multicellular green alga *Volvox carteri*, RegA is involved in somatic cell differentiation (Kirk et al., 1999), while the DEAF-1 protein is necessary for embryonic development in *Drosophila melanogaster* (Veraksa et al., 2002), and its ortholog in mammals is involved in breast epithelial cell differentiation (Barker et al., 2008). In addition, AIRE is an important transcriptional activator to regulate autoimmune processes in the thymus (Abramson et al., 2010).

In plants, ULT1 functions have been described only for Arabidopsis and rice (see below); however, several ULT1 sequences have been reported in other species. In this kingdom, ULT1 seems to be a protein exclusive to Angiosperms, since Gymnosperm, Lycophytes or Mosses lack sequences homologous to ULT1. In angiosperms ULT1 is highly conserved in different species of Eudicots, Monocotyledons, and even in Amborellales, considered one of the most basal angiosperms (Chase et al., 2016), the latter being closer to Eudicots than to Monocotyledons (Figure 2B). The topology of neighbor-joining phylogenetic analysis shows a clear clade distribution according to plant orders, with the exception of *Vitis vinifera* that is closer to Poales (Figure 2B). Evolutionary conservation is also observed for Arabidopsis ULT2, a paralog of ULT1, which conserved a similar protein structure that includes the SAND domain (Carles et al., 2005). The high identity of ULT1 proteins in these species predicts similar functions among them.

## The Role of ULT1 as Part of TrxG Epigenetic Complex

**FIGURE 3:**
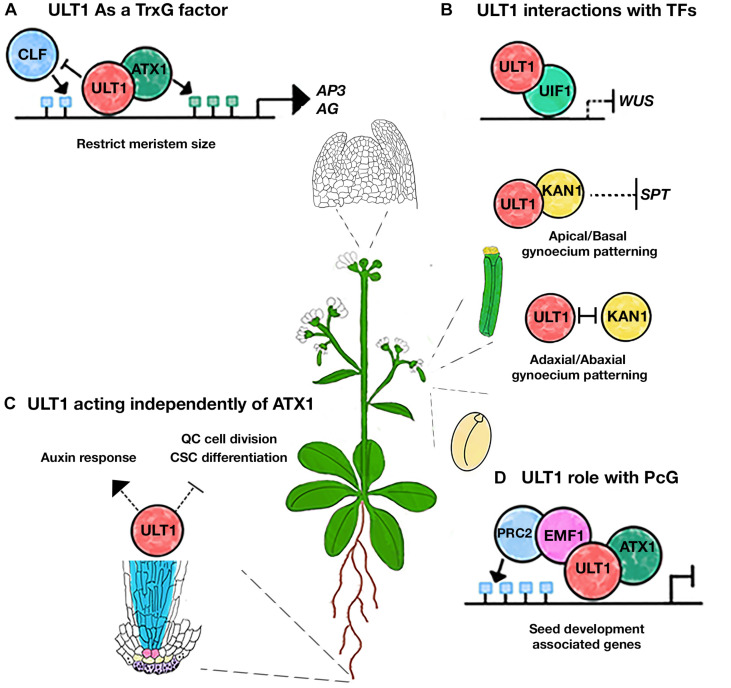
Different roles of ULT1 during Arabidopsis development. **(A)** ULT1 interacts with ATX1 to counteract PcG functions in the SAM. **(B)** ULT1 interacts with UIF1 to repress the *WUS* expression in the SAM. ULT1 may also act with KAN1 to regulate the apical/basal patterning of the gynoecium and it can function antagonistically with KAN1 to regulate the adaxial/abaxial patterning of the gynoecium. **(C)** ULT1 regulates auxin response, the QC cell division rate, and the columella stem cells (CSC) differentiation to maintain root SCN, independently of ATX1. **(D)** ULT1 interacts with EMF1 to keep the repression of seed development genes by maintaining the H3K27me3 marks. The green and blue boxes represent H3K4me3 and H3K27me3 marks, respectively.

The first reports on ULT1 function were made by analyzing the *ULT1* loss and gain-of-function mutant plants (Fletcher, 2001; Carles et al., 2004, 2005; Carles and Fletcher, 2009). Indeed, loss of function of ULT1 delays differentiation and increases shoot and floral meristem size, producing extra-floral organs such as sepals and petals, hence the name ULTRAPETALA (Fletcher, 2001; Carles et al., 2004). In the shoot apical meristem (SAM), ULT1 positively regulates the expression of *APETALA3 (AP3)* and *AGAMOUS (AG)* (Figure 3A), two genes of the ABC flower organ identity model (Carles and Fletcher, 2009). However, ULT1 was also described as a negative regulator of *WUSCHEL (WUS)* expression (Figure 3B), a TF that maintains stem cells in the meristems and must be repressed in order to establish floral determinacy (Carles et al., 2004). Therefore, these reports describe ULT1 as a putative transcriptional regulator, involved in shoot meristem maintenance and floral meristem differentiation and determinacy. Nevertheless, the opposite regulation between ULT1 and CURLY LEAF (CLF), an HMT of the Arabidopsis PcG repressive complex, observed in some vegetative and reproductive organs (Carles and Fletcher, 2009), as well as the antagonistic function of ULT1 with EMBRYONIC FLOWER1 (EMF1), another PcG component (Pu et al., 2013), together with the ability of ULT1 to physically interact with the ATX1, have led to propose ULT1 as a TrxG factor with coactivator properties of some genes related to the SAM development (Carles and Fletcher, 2009).

Furthermore, despite the lack of HMT activity of ULT1, it has been suggested that *ult1* mutant plants have lower levels of H3K4me3 marks on *AG* and *AP3* genes, which are associated with an increase of H3K27me3 PcG mark on these ULT1 targets (Carles and Fletcher, 2009; Pu et al., 2013), evidencing the ability of ULT1 to regulate these epigenetic marks. Interestingly, the 5′-GAGAG-3′ Arabidopsis PRE motifs recognized by CLF and its functional homolog SWINGER (SWN), as well as by other core components of PcG (Deng et al., 2013; Xiao et al., 2017; Shu et al., 2019), can also be recognized by the OsULT1 SAND-domain (Roy et al., 2019). Given that the ULT1 SAND-domains from rice and Arabidopsis share 90.91% of similarity (Figure 2A), it could be predicted that Arabidopsis ULT1 can bind through its SAND domain to the same sites as PcG proteins and thereby interfere with H3K27me3 marks.

All of these reports indicate that ULT1 is a unique SAND-domain protein that is part of a TrxG complex; neither in animals nor in yeast is there evidence of SAND-domain proteins in the TrxG complexes described so far.

## Different Tissues, Different ULT1 Mechanisms

Although ULT1 is able to bind to ATX1, its interactions with other TrxG components are unclear. Unlike the other members of the TrxG, ULT1 has a very discrete expression pattern, being mainly expressed in young organ primordia and shoot and root meristems (Carles et al., 2005; Ornelas-Ayala et al., 2020). This suggests that ULT1 has a tissue-specific regulation rather than a general expression pattern as do the other TrxG members.

Genome-wide analyses have revealed that ULT1-regulated genes are involved in different developmental processes (Tyler et al., 2019). Besides its function in SAM development (Fletcher, 2001), ULT1 participates in different stress processes (Pu et al., 2013; Tyler et al., 2019). In addition, we recently found that ULT1 is necessary for root stem cell niche (SCN) maintenance (Figure 3C), including the cell division rate of the Quiescent Center (QC) and the undifferentiated state of the columella stem cells (Ornelas-Ayala et al., 2020). Interestingly and in contrast to its role in the SAM, our genetic analyses of *atx1* and *ult1* single and double mutants revealed that in the root apical meristem (RAM) ULT1 acts independently of ATX1 (Ornelas-Ayala et al., 2020). The *ult1* mutants showed a diminished response to auxins, demonstrated by a down regulation of some efflux *PIN* transporter genes and the *DR5-GUS* reporter, as well as a premature columella stem cell differentiation (Ornelas-Ayala et al., 2020). Contrary to this, *atx1* mutants do not seem to have defects in auxin response, whereas the columella stem cell differentiation seems to be delayed; besides, in contrast to *atx1* mutants, *ult1* plants did not show any changes in the root and RAM length (Napsucialy-Mendivil et al., 2014; Ornelas-Ayala et al., 2020).

Although the studies of the relationship between ULT1 and ATX1 in the SAM were carried out by single mutant analysis and biochemical methods, and in the RAM were carried out by genetic analysis of double mutants, with these studies, it is possible to establish that ULT1 can act by different mechanisms in the SAM and in the RAM, one of which requires ATX1 to regulate some aspects of floral development while in the other, ULT1 maintains SCN homeostasis in ATX1-independent manner.

In this regard, 18.7% (2859) of Arabidopsis genes are deregulated in *atx1* loss-of-function mutants, whereas 5.6% (856) are deregulated in *ult1* mutants, and among them only a little subset is shared (1.1%; 170 genes) in both *atx1* and *ult1* mutants (Xu et al., 2018); although this does not mean that it is a direct regulation by ATX1 or ULT1, it reflects the behavior of genes that do not always act together. In fact, by ChIP-seq analysis, it has also been determined that out of the 2,276 Arabidopsis TFs annotated (Perez-Rodriguez et al., 2010; Jin et al., 2017), ATX1 is bound to 43 (1.88%) of these, whereas ULT1 to 67 (2.9%) and only in 18 (0.8%) of these are bound both ATX1 and ULT1 (Xu et al., 2018), evidencing that ATX1 and ULT1 have independent targets.

The ATX1-independent function of ULT1 raises the question whether ULT1 acts together with other HMTs of the TrxG complex or by a TrxG-independent mechanism or both in different developmental processes. The analysis of ULT1 protein interactions in different developmental contexts could provide evidence compatible with both mechanisms as shown below.

## ULT1 Acts Together With Some Transcription Factors

The presence of the B-box motif in ULT1 suggests multiple interactions with other proteins. Indeed, ULT1 interacts with some TFs (Figure 3B). One of these is the GARP family transcription factor KANADI1 (KAN1), described as a transcriptional repressor, involved in the patterning of the abaxial polarity of leaves and the gynoecium (Eshed et al., 2001; Pires et al., 2014; Xie et al., 2015). ULT1 interacts physically with KAN1 and genetic analysis indicates that they participate together in the apical-basal polarity of the gynoecium, restricting the *SPATULA (SPA)* expression, which promotes carpel marginal tissue apical style and stigma tissue formation (Figure 3B). But also, ULT1 and KAN1 may act antagonistically to regulate the adaxial-abaxial axis of the gynoecium (Pires et al., 2014; Figure 3B). ULT2 also physically interacts with KAN1, performing redundant roles on the apical-basal gynoecium patterning (Monfared et al., 2013; Pires et al., 2014).

Furthermore, the physical interaction of ULT1 with the MYB domain-containing TF ULTRAPETALA INTERACTING FACTOR 1 (UIF1) has been reported. UIF1 binds to *WUS* and *AG* regulatory sequences in the floral meristem (Moreau et al., 2016). Given that UIF1 acts as a transcriptional repressor, it has been suggested that it represses *WUS* expression when interacting with ULT1, to establish floral meristem determinacy (Moreau et al., 2016; Figure 3B).

These reports have led to suggestions that ULT1 can act as a link between chromatin-remodeling factors and some TFs (Pires et al., 2014). However, other evidence will be needed to indicate whether the combined function of ULT1 with these TFs depends on the other components of TrxG or is TrxG-independent.

## Can ULT1 Act in Different TrxG Complexes?

The lower levels of H3K4me3 marks detected in some genes in the *ult1* mutants compared with those observed in *atx1* mutants (Xu et al., 2018) support the idea that ULT1 can act together with TrxG complex but independently of ATX1, suggesting the existence of different TrxG complexes, through which ULT1 can perform its function. In this regard, multiple SET or MLL HMT homologues from yeasts and animals that can form different COMPASS-like complexes and predict the existence of different TrxG complexes in plants (Schuettengruber et al., 2011). The Arabidopsis compass-like complex reported so far contains ATX1 as the H3K4me3 HMT (Jiang et al., 2009, 2011); however, there are other HMTs of H3K4, such as ATX1/SDG27, ATX2/SDG30, ATXR3/SDG2 and ATXR7/SDG25, that could form different COMPASS complexes (Sanchez et al., 2015). Indeed, it has been demonstrated that the SAND domain of OsULT1 is responsible for interacting with the SET-domain of OsTRX1, an ATX1 ortholog (Roy et al., 2019). The high similarity of Arabidopsis and rice SAND-domains of ULT1 (Figure 2A) suggests that ULT1 can also interact with different proteins with a SET-domain.

Of particular interest is ATXR3/SDG2, reported as the main HMT of the Arabidopsis (Guo et al., 2010). ATXR3/SDG2 does not have a significant sequence homology with other SDGs outside of the SET domain. However, the gene encoding this protein is broadly expressed and is crucial for multiple Arabidopsis developmental processes, regulating 46.4% of all H3K4me3 sites in the Arabidopsis genome (Berr et al., 2010; Guo et al., 2010; Chen et al., 2017). In root tissues, the *sdg2* loss-of-function mutant shares some phenotypes with *ult1* mutants, such as disorganization of the SCN, early differentiation of the columella stem cells, and diminished auxin response (Yao et al., 2013; Ornelas-Ayala et al., 2020). Although it is still unknown whether ULT1 interacts with SDG2, the similarities in their phenotypes raises the possibility that ULT1 could act with SDG2 in some developmental contexts.

## Does ULT1 Function as a Molecular Epigenetic Switch?

Besides the interactions with TFs and TrxG factors, ULT1 also interacts with EMF1 (Xu et al., 2018). EMF1 is the plant-specific protein proposed as a component of Polycomb repressive complex 1 (PRC1), acting as a bridge to the Polycomb repressive complex 2 (PRC2) (Calonje et al., 2008; Wang et al., 2014). Although the relevance of such interaction is unknown, the H3K27me3 abundancy on some EMF1-target genes associated with seed development decreases more in the *emf1/ult1/atx1* triple mutant than in *emf1*, *atx1*, or *ult1* single mutant (Xu et al., 2018). In this framework, it has been proposed that ULT1 interacts with ATX1 to form a complex with PRC2 through EMF1 to maintain the H3K27me3 marks and a chromatin repressive state (Xu et al., 2018). This model suggests that ULT1 not only acts to antagonize the PcG activity; instead, it could act together with PRC2, maintaining the repression states of some targets, through the maintenance of the H3K27me3 mark (Figure 3D). For instance, it has been seen that the *ult1* mutants have more upregulated genes than down-regulated genes (Xu et al., 2018; Tyler et al., 2019). Interestingly, the MADS-box *FLOWERING LOCUS C (FLC)* gene, which is activated by TrxG and repressed by PcG (Whittaker and Dean, 2017), is upregulated (∼4.35 fold) in *ult1* mutant plants (Pu et al., 2013; Xu et al., 2018; Tyler et al., 2019), contrary to what is expected for TrxG mutants. Besides, ULT1 binding to the *FLC* locus supports a direct regulation (Xu et al., 2018). Moreover, the *FLC* upregulation is higher in *ult1/emf1* double mutants than in the *emf1* single mutant (Pu et al., 2013). Hence, loss of *ULT1* function enhances *emf1* upregulation on *FLC*. In contrast, a different behavior was observed on genes that are positively regulated by ULT1, e.g., *AG*, whose upregulation in *emf1* loss-of-function mutants is abated in the double mutant *ult1/emf1* plants (Pu et al., 2013). Although additional experiments are needed, these observations support the involvement of ULT1 in transcriptional repression. Moreover, the repressive function of ULT1 could be compatible with *WUS* repression via UIF1 (Moreau et al., 2016), where PcG could also be participating, as it has been reported (Xu and Shen, 2008).

Given these observations, we suggest two modes of ULT1 action: one through TrxG to regulate transcriptional activation via H3K4me3 deposition, which can be ATX1 dependent or independent, and another, through PcG via EMF1 to repress transcription.

The apparent dual function of ULT1 has led us to wonder whether ULT1 can act as a molecular epigenetic switch, regulating transcriptional repression and activation via PcG and TrxG, respectively. The presence of molecular epigenetic switches allows a dynamic regulation, capable of changing gene expression quickly and efficiently to face different environmental and developmental states. The existence of bivalent chromatin domains provides persuasive evidence of molecular epigenetic switches that regulate gene expression (Hoffmann et al., 2015). The bivalent domains produced by TrxG and PcG serve to keep developmental genes on standby, primed for subsequent expression and to protect against unscheduled expression, reducing transcriptional noise in favor of robust developmental decisions (Hoffmann et al., 2015). Although in plant biological studies, bivalent marks in the same locus have been little addressed and still remain elusive, finding proteins involved in both activation and repression processes shows the relevance of bivalent marks to regulating gene expression quickly and efficiently. In this regard, ULT1 fulfills the main features to act as a molecular epigenetic switch: (i) interaction with both TrxG and PcG proteins, (ii) the ability to increase or decrease gene expression, and (iii) the ability to regulate the deposition of H3K4me3 and H3K27me3 marks. However, establishing whether these characteristics converge into specific genes in time and/or space is still necessary, in such a way that ULT1 can be a link to load the TrxG or PcG complexes and consequently regulate gene expression accordingly.

## Conclusion and Perspectives

Current knowledge reveals ULT1 to be a versatile protein able to interact with TFs, TrxG, and PcG proteins to regulate gene expression of several developmental processes: (1) ULT1 activates genes related to floral development through its interaction with ATX1, (2) in association with UIF1, ULT1 represses *WUS* expression to regulate shoot and floral meristem homeostasis, (3) ULT1 is also involved in the regulation of gynoecium patterning, in which it interacts with KAN1 to repress *SPT*, (4) ULT1 together with EMF1 maintains repressive marks of some genes related to seed development, and (5) ULT1, independently of ATX1, is involved in the root SCN maintenance (Figure 3). The ability of ULT1 to regulate both gene expression and repression by modulation of H3K4me3 and H3K27me3 bivalent marks makes this protein a suitable candidate to regulate bivalent genes that can be in a poised state, waiting for future instructions from the cell. The role of ULT1, independent of ATX1 in roots tissues, suggests a function with other TrxG factors, evidencing the possible existence of different TrxG complexes that could be formed in a tissue-specific fashion in which ULT1 could be involved.

The complexity of ULT1 interactions, the phenotypes reported for *ult1* mutants, and their genome-wide effects make it difficult to define modes of action of ULT1. However, these reports illustrate four possible ways of action for ULT1: (i) together with TrxG factors, (ii) with PcG factors, (iii) outside of both TrxG/PcG complex, and (iv) in association with TFs. Furthermore, a possible mechanism cannot be ruled out through which ULT1 and TrxG or PcG converge in association with TFs.

It would be important to study specific ULT1 targets in different developmental and/or tissue-specific stages to analyze the ULT1 involvement on its activation or repression, which could shed light on the role of ULT1 in association with TrxG and PcG complexes, as a molecular epigenetic switch. Therefore, the future challenge is to define whether ULT1 acts by different mechanisms or in a single mechanism that involves all reported interactions. In this regard, additional research is needed to define whether these mechanisms can coexist or are tissue-, cell type-, or loci-specific.

## Author Contributions

DO-A and MPS conceived and wrote the review. AG-A, ERA-B, and BG-P wrote the review. All authors have read and approved this version of the manuscript.

## Conflict of Interest

The authors declare that the research was conducted in the absence of any commercial or financial relationships that could be construed as a potential conflict of interest.
